# Guideline concordance and antibiotic-associated adverse events between Veterans administration and non-Veterans administration dental settings: a retrospective cohort study

**DOI:** 10.3389/fphar.2024.1249531

**Published:** 2024-01-16

**Authors:** Swetha Ramanathan, Charlesnika T. Evans, Ronald C. Hershow, Gregory S. Calip, Susan Rowan, Colin Hubbard, Katie J. Suda

**Affiliations:** ^1^ School of Public Heath, University of Illinois at Chicago, Chicago, IL, United States; ^2^ Center of Innovation for Complex Chronic Healthcare, Hines VA Hospital, Hines, IL, United States; ^3^ Department of Preventive Medicine and Center for Health Services and Outcomes Research, Northwestern University of Feinberg School of Medicine, Chicago, IL, United States; ^4^ College of Pharmacy, University of Illinois at Chicago, Chicago, IL, United States; ^5^ College of Dentistry, University of Illinois at Chicago, Chicago, IL, United States; ^6^ Division of Hospital Medicine, Department of Medicine, University of California San Francisco, San Francisco, CA, United States; ^7^ Center for Health Equity Research and Promotion, VA Pittsburgh Health Care System, Pittsburgh, PA, United States; ^8^ Department of Medicine, University of Pittsburgh, Pittsburgh, PA, United States

**Keywords:** antibiotic, dentist, adverse drug event, medication safety, prescribing patterns

## Abstract

**Background:** Antibiotics prescribed as infection prophylaxis prior to dental procedures have the potential for serious adverse drug events (ADEs). However, the extent to which guideline concordance and different dental settings are associated with ADEs from antibiotic prophylaxis is unknown.

**Aim:** The purpose was to assess guideline concordance and antibiotic-associated ADEs and whether it differs by VA and non-VA settings.

**Methods:** Retrospective cohort study of antibiotic prophylaxis prescribed to adults with cardiac conditions or prosthetic joints from 2015 to 2017. Multivariable logistic regression models were fit to assess the impact of ADEs, guideline concordance and dental setting. An interaction term of concordance and dental setting evaluated whether the relationship between ADEs and concordance differed by setting.

**Results:** From 2015 to 2017, 61,124 patients with antibiotic prophylaxis were identified with 62 (0.1%) having an ADE. Of those with guideline concordance, 18 (0.09%) had an ADE while 44 (0.1%) of those with a discordant antibiotic had an ADE (unadjusted OR: 0.84, 95% CI: 0.49–1.45). Adjusted analyses showed that guideline concordance was not associated with ADEs (OR: 0.78, 95% CI: 0.25–2.46), and this relationship did not differ by dental setting (Wald χ^2 *p*-value for interaction = 0.601).

**Conclusion:** Antibiotic-associated ADEs did not differ by setting or guideline concordance.

## Introduction

A concern with dental antibiotic prophylaxis is the potential for adverse drug events (ADEs) [e.g., *Clostridioides difficile* infection (CDI)] (Becker, 2014; Ouanounou et al., 2020). Knowledge of patient medical history is vital to help prevent ADEs, however, most health information in dental clinics is self-reported by the patient and not through medical clinician notes in the electronic health record (EHR) (Jones et al., 2017). The Department of Veterans Affairs is the largest integrated healthcare system in the US and incorporates an integrated EHR. This EHR integration is rare in private sector dentistry (Rudman et al., 2010). However, data is scarce whether access to an integrated EHR can prevent antibiotic-associated ADEs by facilitating dentists’ access to risk factors for ADEs contained in the medical record. In addition, little is known if prescribing antibiotic prophylaxis consistent with guidelines mitigates the risk of ADEs. The most recent guidelines on the use of antibiotic prophylaxis to prevent infective endocarditis (IE) and prosthetic joint infection (PJI) were released in 2007 and 2013, respectively (Rethman et al., 2013). Current guidelines from the American Heart Association (AHA) recommend use of antibiotic prophylaxis to prevent IE in patients with specific cardiac conditions undergoing invasive dental procedures (Wilson et al., 2007). Guidelines from the American Academy of Orthopedic Surgeons (AAOS) and the American Dental Association (ADA) do not recommend the routine use of antibiotics for prevention of PJI among those with prosthetic joints (Rethman et al., 2013).

A systematic review and meta-analysis by Cahill et al. (2017) showed that antibiotic prophylaxis is effective in reducing the incidence of bacteremia. However, this may not translate to incidence of IE. Observational studies suggest that antibiotic prophylaxis is not protective with the exception of patients with cardiac conditions at high risk of an adverse outcome if infective endocarditis does occur (Thornhill et al., 2023b; 2022b). Regardless, Cahill and others have concluded that the evidence base for use of antibiotic prophylaxis for IE is limited and has significant limitations (Cahill et al., 2017; Suda et al., 2023). Furthermore, despite recommendations and evidence that suggest that antibiotic prophylaxis for PJIs is not necessary (Rethman et al., 2013; Thornhill et al., 2023a; 2022a), dentists continue to prescribe antibiotics for patients with prosthetic joints. A survey sent to dentists showed that though 95% followed the AHA guidelines for IE, guidelines were not followed in patients with prosthetic joint replacements (Spittle et al., 2017). The survey identified that 72% of dentists prescribed antibiotics within the first 2 years after joint replacement (the highest risk period for the development of PJI) and 58% continue to prescribe beyond 2 years. These findings are particularly important as antibiotic prophylaxis is not without risk. Thornhill et al. (2015) identified that even short antibiotic treatments associated with IE prophylaxis resulted in adverse drug reactions, including *C. difficile* infection (CDI). Therefore, the goal of this study was to assess an association between guideline concordance and antibiotic-associated ADEs and evaluate whether it differs by VA (with an integrated EHR) and non-VA dental settings. As the VA has access to medical data, we hypothesize that VA patients will have less ADEs associated with antibiotic prophylaxis compared to non-VA patients.

## Materials and methods

### Study design, study population and data sources

This was a retrospective cohort study of Veterans and non-Veterans with dental antibiotic prescriptions/visits from 1 January 2015, through 31 December 2017. The cohort included adults (18+) with a cardiac condition or prosthetic joint, identified using ICD-9/10 codes. Veteran data was obtained from the Corporate Data Warehouse. Non-Veteran data was obtained from the IBM Health Marketscan Commercial Claims/Encounters, Medicare Supplemental, Coordination Benefits and IBM Health Dental Claims. Data extraction and analysis were conducted between 2019 and 2022. This study received approval from the Edward Hines, Jr. Institutional Review Board and the University of Illinois at Chicago Institutional Review Board.

### Variable definitions

The main independent variable was guideline concordance defined as a prescription for an antibiotic with a days’ supply of ≤3 days for a patient with a cardiac condition (prosthetic values/material, history of infective endocarditis, cardiac transplant, congenital heart disease) undergoing a dental procedure warranting antibiotic prophylaxis (e.g., gingival manipulation or perforation of the oral mucosa) according to the AHA guidelines (Wilson et al., 2007). Antibiotics prescribed to patients with prosthetic joints was defined as guideline discordant according to AAOS/ADA guidelines (Rethman et al., 2013). The primary outcome variable (“ADEs”) was a composite indicator variable for the occurrence of anaphylaxis and antibiotic-related allergic reactions (within 14 days of antibiotic receipt), or CDI (within 30 days of antibiotic receipt), identified using ICD-9 and ICD-10 codes (Gross et al., 2019b).

Additional covariates included dental setting (VA/Non-VA), age (18–34, 35–44, 45–54, 55–64, and ≥65 years), gender (Male/Female), US geographic region (North, Midwest, South, West), location (Urban/Rural), and antibiotic agent [amoxicillin, clindamycin, cephalexin, azithromycin, penicillin, doxycycline, other (e.g., fluroquinolones)]. Prescriptions for amoxicillin/clavulanate were grouped with amoxicillin. Antibiotics were analyzed as dichotomous variables (Yes vs. No). Dental procedures were identified using the American Dental Association’s Code on Dental procedures and Nomenclature (CDT) codes and grouped into categories of service and analyzed as a dichotomous variable (Received vs. Did not receive procedure). Comorbid conditions were assessed using the composite Charlson Comorbidity index and the individual clinical conditions that are elements of the index, identified using ICD-9/10 codes.

### Statistical analysis

The frequency distribution of ADEs was examined using Student’s *t*-test and Pearson Chi-square tests. Multivariable logistic regression was conducted with covariates selected based on statistical significance (*p* < 0.05) and epidemiological consideration. The final model was selected using backwards selection, using the Akaike Information Criterion (AIC). Additionally, a term modeling the potential interaction of guideline concordance and dental setting was included in the multivariable model and the Wald chi-square test was used to evaluate if the relationship between downstream outcomes and concordant prescribing differed by setting. All analyses were conducted with STATA software version 14.2 (StataCorp, College Station, TX).

## Results

From 2015 to 2017, 61,124 patients with antibiotic prophylaxis prescriptions were identified with 20,014 (32.7%) defined as guideline concordant and 62 (0.1%) having an ADE (61.3% of ADEs reported in non-VA settings and 38.7% of ADEs reported in VA settings). There were 42 people with an allergic reaction, 1 with anaphylaxis, and 19 with CDI. Of those with guideline concordance, 18 (0.09%) had an ADE while 44 (0.1%) of those with guideline discordance had an ADE (unadjusted OR: 0.84, 95% CI: 0.49–1.45). Comorbidities increased the odds of an ADE while those with diagnostic or preventive procedures had decreased odds of ADEs (Table 1).

**TABLE 1:** Distribution of characteristics by adverse drug events among those with cardiac conditions or prosthetic joints receiving a dental antibiotic prescription.

Variables	Total N = 61,124	ADE (%) N = 62	No ADE (%) N = 61,062	Chi square *p*-value	Unadjusted odds ratio (95% CI)
*Main independent variable*					
Guideline concordant					
No	41,110 (67.3)	44 (71.0)	41,066 (67.3)	0.533	Reference
Yes	20,014 (32.7)	18 (29.0)	19,996 (32.7)		0.84 (0.49–1.45)
*Demographics and clinical characteristics*					
Age	59.1 (10.4); [18,99]	59.0 (11.1)	59.1 (10.4)	0.956	0.99 (0.98–1.02)
Age group					
18–24	1,799 (2.9)	4 (6.5)	1,795 (2.9)	0.500	Reference
35–44	2,361 (3.9)	3 (4.8)	2,358 (3.9)		0.57 (0.13–2.55)
45–54	10,382 (17.0)	8 (12.9)	10,374 (17.0)		0.35 (0.10–1.15)
55–64	33,641 (55.0)	34 (54.8)	33,607 (55.0)		0.45 (0.16–1.28)
65+	12,941 (21.2)	13 (21.0)	12,928 (21.2)		0.45 (0.15–1.39)
Sex					
Male	37,253 (61.0)	34 (54.8)	37,219 (61.0)	0.324	Reference
Female	23,871 (39.0)	28 (45.2)	23,843 (39.1)		1.29 (0.78–2.12)
Cardiac condition					
No	38,445 (62.9)	40 (64.5)	38,405 (62.9)	0.792	Reference
Yes	22,679 (37.1)	22 (35.5)	22,657 (37.1)		0.93 (0.55–1.57)
Prosthetic joint					
No	15,707 (25.7)	11 (17.7)	15,696 (25.7)	0.152	Reference
Yes	45,427 (74.3)	51 (82.3)	45,366 (74.3)		1.60 (0.84–3.07)
Cardiac condition or prosthetic joint					
Cardiac condition	15,707 (25.7)	11 (17.7)	15,696 (25.7)	0.154	Reference
Prosthetic joint	38,445 (62.9)	40 (64.5)	38,405 (62.9)		1.49 (0.76–2.90)
Both	6,972 (11.4)	11 (17.7)	6,961 (11.4)		2.25 (0.98–5.20)
Region					
Northeast	7,901 (12.9)	14 (22.6)	7,887 (12.9)	0.041	Reference
Midwest	22,780 (37.3)	25 (40.3)	22,755 (37.3)		0.62 (0.32–1.19)
South	22,635 (37.0)	14 (22.6)	22,621 (37.1)		0.35 (0.17–0.73)
West	7,808 (12.8)	9 (14.5)	7,799 (12.8)		0.65 (0.28–1.50)
Location					
Rural	9,659 (15.8)	11 (17.7)	9,648 (15.8)	0.675	Reference
Urban	51,465 (84.2)	51 (82.3)	51,414 (84.2)		0.87 (0.45–1.67)
Dental setting					
Non-VA	42,832 (70.1)	38 (61.3)	42,794 (70.1)	0.131	Reference
VA	19,292 (29.9)	24 (38.7)	18,268 (29.9)		1.48 (0.88–2.47)
Year					
2015	20,684 (33.8)	18 (29.0)	20,666 (33.8)	0.262	Reference
2016	20,745 (33.9)	18 (29.0)	20,727 (33.9)		0.99 (0.52–1.92)
2017	19,695 (32.2)	26 (42.0)	19,669 (32.3)		1.52 (0.83–2.77)
*Comorbidities*					
Charlson comorbidity index	0.91 (1.5) [0,17]	2.3 (2.8)	0.9 (1.5)	<0.001	**1.35 (1.24–1.47)**
Myocardial infarction					
No	60,217 (98.5)	59 (95.2)	60,158 (98.5)	0.029	Reference
Yes	907 (1.5)	3 (4.8)	904 (1.5)		**3.38 (1.05–10.81)**
Congestive heart failure					
No	56,315 (92.1)	52 (83.9)	56,263 (92.1)	0.016	Reference
Yes	4,809 (7.9)	10 (16.1)	4,799 (7.9)		**2.25 (1.15–4.44)**
Peripheral vascular disease					
No	57,330 (93.8)	54 (87.1)	57,276 (93.8)	0.029	Reference
Yes	3,794 (6.2)	8 (12.9)	3,786 (6.2)		**2.24 (1.07–4.71)**
Cerebrovascular disease					
No	58,824 (96.2)	55 (88.7)	58,769 (96.2)	0.002	Reference
Yes	2,300 (3.8)	7 (11.3)	2,293 (3.8)		**3.26 (1.48–7.17)**
Dementia					
No	60,811 (99.5)	60 (96.8)	60,751 (99.5)	0.003	Reference
Yes	313 (0.5)	2 (3.2)	311 (0.5)		**6.51 (1.58–26.76)**
COPD					
No	53,515 (87.6)	44 (71.0)	53,471 (87.6)	<0.001	Reference
Yes	7,609 (12.4)	18 (29.0)	7,591 (12.4)		**2.88 (1.66–4.99)**
Connective tissue disease					
No	59,172 (96.8)	58 (93.6)	59,114 (96.8)	0.144	Reference
Yes	1,952 (3.2)	4 (6.4)	1,948 (3.2)		2.09 (0.76–5.77)
Peptic ulcer disease					
No	60,657 (99.2)	59 (95.2)	60,598 (99.2)	<0.001	Reference
Yes	467 (0.8)	3 (4.8)	464 (0.8)		**6.64 (2.07–21.26)**
Liver disease					
No	59,063 (96.6)	57 (91.9)	59,006 (96.6)	0.064	Reference
Mild	1,534 (2.5)	3 (4.8)	1,531 (2.5)		2.03 (0.63–6.48)
Moderate to severe	527 (0.9)	2 (3.2)	525 (0.9)		3.94 (0.96–16.20)
Diabetes					
No	51,932 (85.0)	45 (72.6)	51,887 (85.0)	<0.001	Reference
Uncomplicated	6,880 (11.3)	8 (12.9)	6,872 (11.3)		1.34 (0.63–2.85)
Complicated	2,312 (3.7)	9 (14.5)	2,303 (3.7)		**4.51 (2.20–9.23)**
Paraplegia/Hemiplegia					
No	60,826 (99.5)	61 (98.4)	60,765 (99.5)	0.203	Reference
Yes	298 (0.5)	1 (1.6)	297 (0.5)		3.35 (0.46–24.27)
Renal disease					
No	56,615 (92.6)	48 (77.4)	56,567 (92.6)	<0.001	Reference
Yes	4,509 (7.4)	14 (22.6)	4,495 (7.4)		**3.67 (2.02–6.66)**
Cancer					
No	57,490 (94.1)	54 (87.1)	57,436 (94.1)	0.020	Reference
Yes	3,634 (5.9)	8 (12.9)	2,626 (5.9)		**2.35 (1.12–4.93)**
Metastatic solid tumor					
No	60,801 (99.5)	62 (100.0)	60,739 (99.5)	0.566	Reference
Yes	323 (0.5)	0 (0.0)	323 (0.5)		—
AIDS					
No	61,035 (99.9)	61 (98.4)	60,974 (99.9)	0.002	Reference
Yes	89 (0.1)	1 (1.6)	88 (0.1)		**11.35 (1.56–82.8)**
*Dental procedures*					
Gingival manipulation					
No	7,505 (12.3)	7 (11.3)	7,498 (12.3)	0.813	Reference
Yes	53,619 (87.7)	55 (88.7)	53,564 (87.7)		1.09 (0.50–2.41)
Adjunctive					
No	57,368 (93.9)	60 (96.8)	57,308 (93.9)	0.338	Reference
Yes	2,756 (6.1)	2 (3.2)	3,754 (6.1)		0.51 (0.12–2.08)
Diagnostic					
No	17,585 (28.8)	25 (40.3)	17,560 (28.8)	0.044	Reference
Yes	43,539 (72.2)	37 (59.7)	43,502 (71.2)		**0.60 (0.36–0.99)**
Endodontics					
No	59,690 (97.7)	60 (96.8)	59,630 (97.7)	0.647	Reference
Yes	1,434 (2.3)	2 (3.2)	1,432 (2.3)		1.39 (0.34–5.68)
Implant					
No	60,268 (98.6)	62 (100.0)	60,206 (98.6)	0.348	Reference
Yes	856 (1.4)	0 (0.0)	856 (1.4)		—
Maxillofacial prosthetics					
No	61,082 (99.9)	62 (100.0)	61,020 (99.9)	0.836	Reference
Yes	42 (0.1)	0 (0.0)	42 (0.1)		—
Oral maxillofacial surgery					
No	57,435 (94.0)	56 (90.3)	57,379 (94.0)	0.228	Reference
Yes	3,689 (6.0)	6 (9.7)	3,683 (6.0)		1.67 (0.72–3.87)
Orthodontics					
No	61,054 (99.9)	62 (100.0)	60,992 (99.9)	0.790	Reference
Yes	70 (0.1)	0 (0.0)	70 (0.1)		—
Periodontics					
No	56,376 (92.2)	56 (90.3)	56,320 (92.2)	0.574	Reference
Yes	4,748 (7.8)	6 (9.7)	4,742 (7.8)		1.27 (0.55–2.95)
Preventive					
No	31,436 (51.4)	40 (64.5)	31,396 (51.4)	0.039	Reference
Yes	29,688 (48.6)	22 (35.5)	29,666 (48.6)		**0.58 (0.35–0.98)**
Removable prosthodontics					
No	59,060 (96.6)	58 (93.6)	59,002 (96.6)	0.180	Reference
Yes	2,064 (3.4)	4 (6.4)	2,060 (3.4)		1.97 (0.72–5.45)
Fixed prosthodontics					
No	60,327 (98.7)	61 (98.4)	60,266 (98.7)	0.830	Reference
Yes	797 (1.3)	1 (1.6)	796 (1.3)		1.24 (0.17–8.96)
Restorative					
No	46,350 (75.8)	44 (71.0)	46,306 (75.8)	0.371	Reference
Yes	14,774 (24.2)	18 (29.0)	14,756 (24.2)		1.28 (0.74–2.22)
Uncategorized					
No	60,717 (99.3)	61 (98.4)	60,656 (99.3)	0.359	Reference
Yes	407 (0.7)	1 (1.6)	406 (0.7)		2.45 (0.34–17.71)
*Antibiotics*					
Amoxicillin					
No	14,723 (24.1)	18 (29.0)	14,705 (24.1)	0.362	Reference
Yes	46,401 (75.9)	44 (71.0)	46,357 (75.9)		0.77 (0.45–1.34)
Clindamycin					
No	52,497 (85.9)	48 (77.4)	52,449 (85.9)	0.055	Reference
Yes	8,627 (14.1)	14 (22.6)	8,613 (14.1)		1.78 (0.97–3.22)
Cephalexin					
No	57,220 (93.6)	59 (95.2)	57,161 (93.6)	0.618	Reference
Yes	3,904 (6.4)	3 (4.8)	3,901 (6.4)		0.75 (0.23–2.38)
Azithromycin					
No	60,378 (98.8)	62 (100.0)	60,316 (98.8)	0.381	Reference
Yes	746 (1.2)	0 (0.0)	746 (1.2)		—
Penicillin					
No	60,737 (99.4)	62 (100.0)	60,675 (99.4)	0.529	Reference
Yes	387 (0.6)	0 (0.0)	387 (0.6)		—
Doxycycline					
No	61,033 (99.8)	61 (100.0)	60,971 (99.9)	0.761	Reference
Yes	91 (0.2)	0 (0.0)	91 (0.1)		—
Other					
No	59,345 (97.1)	60 (96.7)	59,285 (97.1)	0.883	Reference
Yes	1,779 (2.9)	2 (3.2)	1,777 (2.9)		1.11 (0.27–4.55)

Bold values signify significant results.

The multivariable regression model found that guideline concordance was associated with a lowered odds of ADEs (OR: 0.78, 95% CI: 0.25–2.46), though this finding was not statistically significant (Table 2). Patients receiving care from a VA dentist were more likely to experience ADEs (OR = 2.91, 95% CI 1.37–6.15). Furthermore, there was no evidence that the relationship between guideline concordance and ADEs was modified by dental setting in multivariable models (Wald $\chi^{2}$ *p*-value = 0.601).

**TABLE 2:** Multivariable model for adverse drug events among those with cardiac conditions or prosthetic joints receiving a dental antibiotic prescription.

Variables	Odds ratio (95% CI)	Wald $\chi^{2}$ *p*-value
Guideline concordance (Yes vs. No)	0.78 (0.25–2.46)	
Dental setting (VA vs. Non-VA)	**2.91 (1.37–6.15)**	0.601
Age group		
35–44 vs. 18–24	0.40 (0.09–1.83)	
45–54 vs. 18–24	0.18 (0.05–0.64)	
55–64 vs. 18–24	0.20 (0.06–0.60)	
65+ vs. 18–24	0.11 (0.03–0.42)	
Sex (Female vs. Male)	**1.88 (1.02–3.45)**	
Cardiac condition or prosthetic joint		
Prosthetic joint vs. Cardiac condition	1.91 (0.58–6.28)	
Both vs. Cardiac condition	2.38 (0.97–5.81)	
Region		
Midwest vs. Northeast	0.63 (0.33–1.22)	
South vs. Northeast	**0.32 (0.15–0.68)**	
West vs. Northeast	0.63 (0.27–1.47)	
Charlson	**1.37 (1.26–1.50)**	
Diagnostic (Yes vs. No)	0.64 (0.38–1.09)	

Bold values signify significant results.

## Discussion

This is the first study to compare ADEs related to guideline concordance of antibiotic prophylaxis by VA and non-VA dental settings. There was no association between guideline concordance and ADEs, indicating that any use of antibiotic prophylaxis regardless of appropriateness is associated with risk. There was also no difference in the occurrence of ADEs by dental setting. Thus, our hypothesis, that VA dentists’ access to a nationally integrated EHR may facilitate the identification of risk factors for antibiotic-associated ADEs (e.g., history of CDI) was rejected. Potential explanation for this finding may be that VA and non-VA patients differed in characteristics. While we attempted to adjust for age, co-morbidities (through the Charlson score), and dental procedures, differences between populations may remain. For example, past exposure to antibiotics or history of ADEs was not assessed.

While the overall incidence was low, serious antibiotic-associated ADEs, such as CDI and antibiotic allergies, can be life-threatening. Gross et al. (2019b) identified 1.4% of a cohort with commercial insurance receiving unnecessary antibiotic prophylaxis had an ADE which is larger than our findings. However, Gross included ED visits and used a 4-year study period (vs. 3 years herein) (Gross et al., 2019b). A prospective study found that 1.5% of patients developed an adverse reaction with 0.9% reporting rash and 0.5% reporting diarrhea after receipt of a single dose of a prophylactic antibiotic (Sandrowski et al., 2020). Prior work in VA found antibiotic prophylaxis resulted in 690 (0.9%) CDI cases within 90-days of surgery (Branch-Elliman et al., 2019). A subsequent analysis of VA dental patients found that 0.05% had a positive diagnostic test for *C. difficile* within 30 days of antibiotic prophylaxis (Wilson et al., 2022). Additionally, a retrospective cohort study in Canada found 1.5% of patients who received a pre-operative antibiotic developed CDI (Carignan et al., 2008). Thus, previous studies reveal a range of estimates of the incidence of ADEs, with variation likely deriving from study methodologies (i.e., ICD-9 codes vs. prospective data collection).

To prevent ADEs after dental antibiotic prophylaxis, it is also important to understand why VA patients had a higher odds of ADEs. One reason may be because access to the medical chart allows for better recording of ADEs and/or population differences. A secondary analysis of the National Health Interview Survey of Veterans and those in the general population found that VA patient populations have poorer health, more medical conditions, and higher healthcare resource use compared to the general population (Agha et al., 2000). Assessing data from this study, the VA population was older but the non-VA cohort was slightly sicker. The final model controlled for both age and Charlson within this study. However, the Charlson index does not consider every comorbidity and it is possible that some comorbidities not captured in this model may be driving this result. It is also possible that, despite controlling for age, behavioral or other aspects related to age not accounted for could be influencing this result. For example, older patients may be more likely to follow through with health concerns such as rashes or diarrhea. Alternatively, unique characteristics related to medical setting or VA population may be the reason for this finding. These considerations are discussed in depth below.

Another reason for the differences in ADEs by dental setting is that Veterans may have increased access to care compared to the general population. Eligibility for dental care in the VA is different than other VA benefits (VA dental care, 2020). Those that receive dental care have a set of eligibility criteria in which some individuals have access to any dental care while others have limited access VA dental care (2020). Those that receive any dental care are those with a service-connected dental disability/condition or were a former prisoner of war. Coverage for dental care is very different from those in the non-VA population who can purchase or enroll into commercial dental insurance. Thereby, these VA dental patients may require more frequent and/or complex dental care (i.e., more invasive oral surgeries) than the general population resulting in increased contact with the healthcare system. Therefore, VA patients may be more likely to follow through with concerns such as allergies or CDI than those in the non-VA sector.

Apart from differing dental eligibilities and compensation at VA facilities, VA patients may also have easier access to care. VA patients typically have lower wait times, waiting on average 20 min less for primary care physicians compared to non-VA patients (Penn et al., 2019). Other work published on VA care suggests that VA patients also receive longer appointment times with their physicians (Shulkin, 2016). A combination of lower wait times and increased time with their physician, may motivate more VA patients to seek care with a primary or other clinician types thereby diagnosing more adverse drug events as compared to private sector individuals. Having reliable access to care and lower wait times may make Veterans more likely to follow up with such concerns with healthcare providers.

Current guidelines for antibiotic prophylaxis have been updated, over time, to account for unnecessary overprescribing and have reduced the number of people recommended to receive prophylaxis for IEs and PJIs. Recent guidelines advocate for prophylaxis in select patients with cardiac conditions and recommend no antibiotic prophylaxis for those with prosthetic joints (Wilson et al., 2007; Rethman et al., 2013). Guideline revisions along with antibiotic stewardship may have led to improved prescribing. Previous data have shown that during the time the guidelines were updated in 2007, dental antibiotic prescribing decreased by 38.1% for tetracyclines, 29.9% for cephalosporins, and 13.5% for penicillins with an overall decrease in proportion of prescriptions of 0.7% (from 10.7% in 2005 to 10% in 2010) (Suda et al., 2016). However, more recent data showed that dental prescribing rates remain unchanged between 2012 through 2019, during the AAOS/ADA guideline revisions (Ramanathan et al., 2023). Using administrative claims data, unnecessary antibiotic prophylaxis slightly decreased in a commercially insured population (Hubbard et al., 2022), but increased in the Veteran population (Suda et al., 2022). These results suggest that guideline changes alone may not impact prescribing and antibiotic stewardship should be incorporated into dental settings. While limited, studies have shown that the implementation of antibiotic stewardship strategies can be effective in dental clinics (Gross et al., 2019a; Goff et al., 2022). Consistent with recommendations by the Centers for Disease Control and Prevention, antibiotic stewardship should be a strategy employed in VA and non-VA dental settings as part of a daily clinical practice (The Core Elements of Outpatient Antibiotic Stewardship, 2023).

In order to conduct rigorous medication safety assessments, future research should validate methods to determine antibiotic-associated ADEs. While surveillance datasets (e.g., Medwatch) can be used to identify ADEs, these datasets cannot be linked to other claims or EHR datasets. For example, it is currently difficult to identify an association of unnecessary prescribing and occurrence of an ADE due to limited variables available for analysis in surveillance datasets. Obtaining granular data is essential to determining prevalence and risk factors for ADEs, variables which can be obtained from large claims and EHR databases.

### Limitations and strengths

This study is not without limitations. First, this study could not account for all differences between VA and non-VA settings. Second, we were not able identify the prescribing provider in non-VA data, though analogous approaches were used to identify each cohort and used methods applied in prior work. Ramanathan et al. (2023) Third, these analyses focus on a specific population in VA and non-VA settings, and the results may not be generalizable. Fourth, there could be misclassification as algorithms were used to determine guideline concordance, including undercoding of ICD-9/10s to identify ADEs. Fifth, this study did not account for prior antibiotic exposure, history of ADEs, or indication for prophylaxis which may have influenced the findings. Sixth, the small prevalence of the outcome can result in biased regression estimates, future studies should verify the validity of these findings. Finally, the study could not identify patients who did not seek care for an ADE. However, this study used two of the largest and most comprehensive data sources for dental research. A strength of this study is the first to evaluate downstream adverse events from dental antibiotic prophylaxis in two different dental settings.

## Conclusion

The results of this study provide the foundation for understanding the relationship between dental setting, guideline concordance, and downstream ADEs. The results suggest there is no relationship between guideline concordance of antibiotic prophylaxis and downstream ADEs. Furthermore, this relationship did not differ by VA and non-VA dental settings.

## Data Availability

The data analyzed in this study is subject to the following licenses/restrictions: We are committed to collaborating and sharing these data to maximize their value to improve veterans and others’ health and healthcare, to the greatest degree consistent with current Veterans Health Administration regulations and policy. We can provide access to the programming code used to identify the sample and conduct analyses and will conduct additional analyses as requested. We cannot provide a link to the database directly as it would compromise patients’ anonymity, and permissions from VA are needed to obtain the data. The Marketscan dataset used for this study is proprietary and unable to be shared. Interested individuals may contact the corresponding author (KS) for information. Requests to access these datasets should be directed to KS, ksuda@pitt.edu.
